# *cis*‐Proline mutants of quiescin sulfhydryl oxidase 1 with altered redox properties undermine extracellular matrix integrity and cell adhesion in fibroblast cultures

**DOI:** 10.1002/pro.3537

**Published:** 2018-12-17

**Authors:** Gabriel Javitt, Iris Grossman‐Haham, Assaf Alon, Efrat Resnick, Yael Mutsafi, Tal Ilani, Deborah Fass

**Affiliations:** ^1^ Department of Structural Biology Weizmann Institute of Science Rehovot 7610001 Israel

**Keywords:** oxidoreductase, disulfide, redox potential, *cis*‐proline, extracellular matrix

## Abstract

The thioredoxin superfamily has expanded and diverged extensively throughout evolution such that distant members no longer show appreciable sequence homology. Nevertheless, redox‐active thioredoxin‐fold proteins functioning in diverse physiological contexts often share canonical amino acids near the active‐site (di‐)cysteine motif. Quiescin sulfhydryl oxidase 1 (QSOX1), a catalyst of disulfide bond formation secreted by fibroblasts, is a multi‐domain thioredoxin superfamily enzyme with certain similarities to the protein disulfide isomerase (PDI) enzymes. Among other potential functions, QSOX1 supports extracellular matrix assembly in fibroblast cultures. We introduced mutations at a *cis*‐proline in QSOX1 that is conserved across the thioredoxin superfamily and was previously observed to modulate redox interactions of the bacterial enzyme DsbA. The resulting QSOX1 variants showed a striking detrimental effect when added exogenously to fibroblasts: they severely disrupted the extracellular matrix and cell adhesion, even in the presence of naturally secreted, wild‐type QSOX1. The specificity of this phenomenon for particular QSOX1 mutants inspired an investigation of the effects of mutation on catalytic and redox properties. For a series of QSOX1 mutants, the detrimental effect correlated with the redox potential of the first redox‐active site, and an X‐ray crystal structure of one of the mutants revealed the reorganization of the *cis*‐proline loop caused by the mutations. Due to the conservation of the mutated residues across the PDI family and beyond, insights obtained in this study may be broadly applicable to a variety of physiologically important redox‐active enzymes.

**Impact statement:**

We show that mutation of a conserved *cis*‐proline amino acid, analogous to a mutation used to trap substrates of a bacterial disulfide catalyst, has a dramatic effect on the physiological function of the mammalian disulfide catalyst QSOX1. As the active‐site region of QSOX1 is shared with the large family of protein disulfide isomerases in humans, the effects of such mutations on redox properties, enzymatic activity, and biological targeting may be relevant across the family.

## Abstract 2

PDB Code(s): 6HF1

## Introduction

The thioredoxin (Trx) protein superfamily is characterized by a particular α/β fold and is dominated by members containing the redox‐active Cys‐X‐X‐Cys motif (CXXC), in which X stands for a non‐cysteine amino acid.^1^ The Trx superfamily includes both cytosolic enzymes that function physiologically as disulfide reductants and endoplasmic reticulum‐localized enzymes that function, in part, as oxidants of substrate proteins in that compartment. Both these functions involve nucleophilic attack of a cysteine thiolate on a disulfide to form an intermediate “mixed” disulfide between the enzyme and the substrate, which is then resolved by a second thiolate to yield a new disulfide.^1^ If the identities of the thiolates in the reaction are known, it is straightforward to stabilize the mixed disulfide by disabling the resolving cysteine by mutation. In this manner, enzyme‐substrate complexes can be separated or isolated, and the substrates identified by antibody binding or mass spectrometry.^2^, ^3^, ^4^, ^5^ In contrast, when the participating thiolates are not known, as in the case of enzymes acting as oxidants of as yet unidentified target proteins, this approach cannot be used. The unknown substrate protein contains both the nucleophilic cysteine and the resolving cysteine, while the redox‐active enzyme contains the electrophilic disulfide, which must be preserved to enable mixed disulfide formation. Faced with this problem when searching for substrates of the bacterial Trx superfamily member DsbA, which oxidizes periplasmic proteins, Kadokura et al. found that mutating a conserved *cis*‐proline residue adjacent to the DsbA CXXC motif to threonine stabilized mixed disulfides between DsbA and its targets for oxidation.^6^ Mutation of the same proline to other amino acids favored a mixed disulfide with DsbB, the enzyme that reoxidizes DsbA.^7^

**Figure 1:**
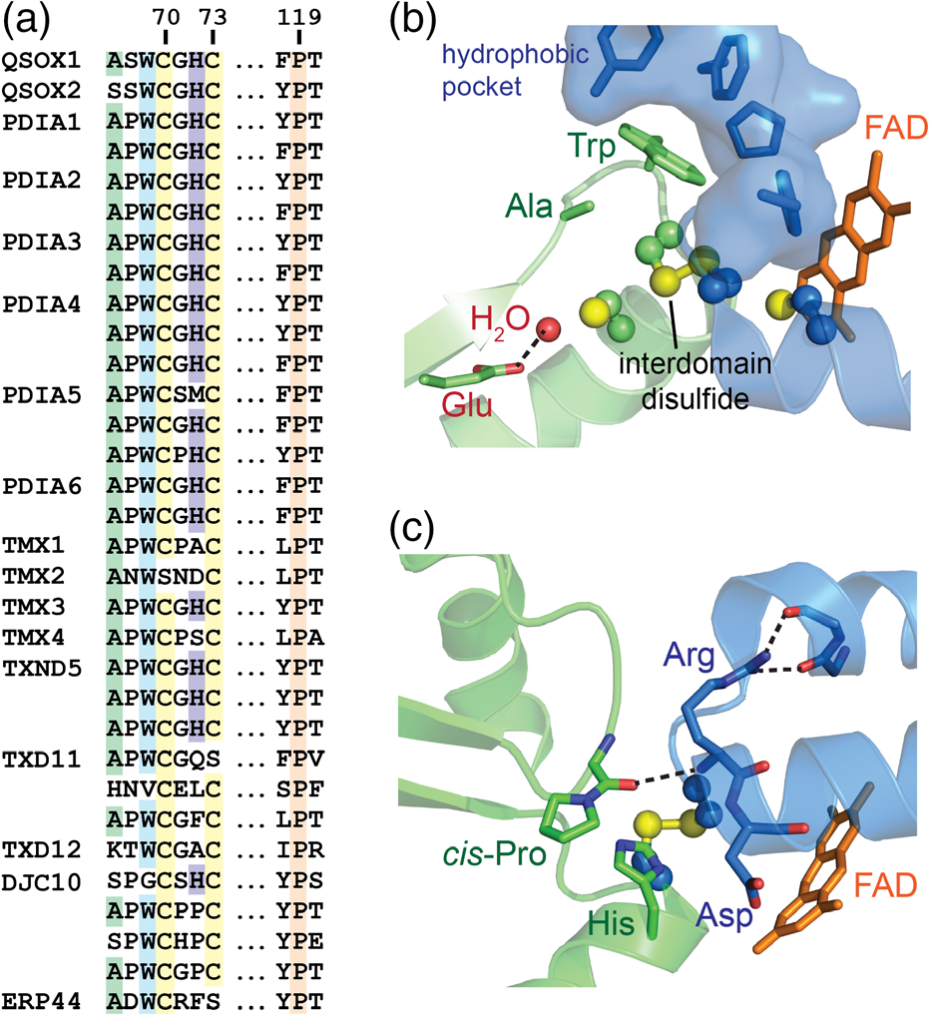
Conserved active‐site motif in PDI family proteins. (a) Amino acid sequence alignment of the CXXC motif and structurally adjacent *cis*‐proline region. Residue numbering corresponds to human QSOX1. Many PDI family proteins have multiple CXXC‐containing domains, which are presented in the order they appear in the protein primary structure. (b) Structure of the interface region between the Trx domain (green) and sulfhydryl oxidase domain (blue) redox‐active motifs of rat QSOX1 (PDB code 4P2L) with an interdomain disulfide bond. The conserved tryptophan (Trp; Residue 69 in human QSOX1) is shown packed in a hydrophobic pocket in the sulfhydryl oxidase domain. The conserved alanine is below the Trp. The isoalloxazine of the flavin adenine dinucleotide cofactor is shown and labeled FAD. A glutamate (Glu) side chain and a buried water molecule (H_2_O) involved in acid–base catalysis are shown. (c) The structure of the interface region from a mutated version of mouse QSOX1 (PDB code 3 T58) containing an interdomain disulfide bond shows the hydrogen bond (dashed line) formed by the backbone carbonyl preceding the *cis*‐proline (*cis*‐Pro). The histidine (His) present in the second X position of many PDI CXXC motifs samples different rotamers in various crystal structures. Here it is shown in the *g* + rotamer (chi1 = ~60°), which places it in proximity to the interdomain disulfide.

The nearly universal conservation of the *cis*‐proline in Trx proteins with known structures^8^ suggests that this mutational approach may be relevant to other superfamily members.^7^ Indeed, a proline is found at the same position in the primary structures of about 30 Trx domains in human Protein Disulfide Isomerase (PDI) family members [Fig. 1(a)]. With the caveat that the presence of a canonical amino acid in a sequence alignment is not a guarantee that the residue actually assumes the standard configuration and performs the recognized function in all cases,^9^ mutation of this proline may confer substrate‐trapping or other useful redox properties to human PDI proteins.

The Quiescin sulfhydryl oxidase (QSOX) enzymes are not generally considered to be PDI family members,^10^ but in fact the first two domains of animal QSOX closely resemble the tandem Trx domains found in PDI proteins.^11^ Similar to PDI itself, the first QSOX domain is redox active and contains a CXXC motif [Fig. 1(a)], whereas the second domain lacks this motif. Both mammalian QSOX orthologs, the predominant QSOX1 and the lower‐abundance QSOX2,^12^ additionally share with the PDI family conserved amino acids in the vicinity of the active‐site cysteines [Fig. 1(a)]. The difference between QSOX enzymes and PDI family proteins is that the QSOX Trx domains are fused to a sulfhydryl oxidase module that internally re‐oxidizes the amino‐terminal Trx domain active site, as demonstrated biochemically^13^, ^14^ and shown in structures detailing the interactions between the PDI‐like and sulfhydryl oxidase domains [Fig. 1(b,c)].^11^, ^15^ In contrast, reduced PDI proteins are reoxidized by intermolecular reactions with sulfhydryl oxidases, PDI peroxidases, or vitamin K epoxide reductase present in the endoplasmic reticulum.^16^ The presence of both the Trx domain active site and a sulfhydryl oxidase module in the same polypeptide as occurs in QSOX enzymes provides a compact system to investigate how mutations in residues near the Trx CXXC motif affect both substrate interactions and the enzyme re‐oxidation process. In this report, we present observations regarding the striking effects of *cis*‐proline and other mutations in QSOX1 on fibroblast cell cultures, in which secreted QSOX1 normally contributes to extracellular matrix (ECM) assembly and functionality.^17^ The findings regarding extracellular activities of QSOX1 mutants are complemented by an investigation of the effects of mutation on redox activity and protein structure, providing information potentially relevant to the many enzymes that share active‐site features with QSOX1.

## Results

### QSOX1 *cis*‐proline mutants disrupt fibroblast cultures

As a tool for uncovering QSOX1 redox interactions in physiological settings, we generated mutants that might prolong the interaction of the enzyme with its substrate(s). By analogy to the corresponding mutants of DsbA,^6^, ^7^ the P119T and P119S mutants of human QSOX1 were produced. In addition, we generated the H72A mutant. H72 was previously observed to assume alternate rotamers in a manner that may be coupled to the configuration of mixed disulfides made by the upstream cysteine in the active site^11^; this histidine may therefore influence the stability of QSOX1 mixed disulfides. The comparable histidine was mutated in a PDI variant used to trap targets in the context of extracellular PDI activity in thrombosis.^18^ QSOX1 mutants containing both H72A and P119T or P119S were also made, and a mutant in which the active‐site Cysteines C70 and C73 were mutated to alanine (C70A/C73A, AXXA variant) was produced as a control.

**Figure 2:**
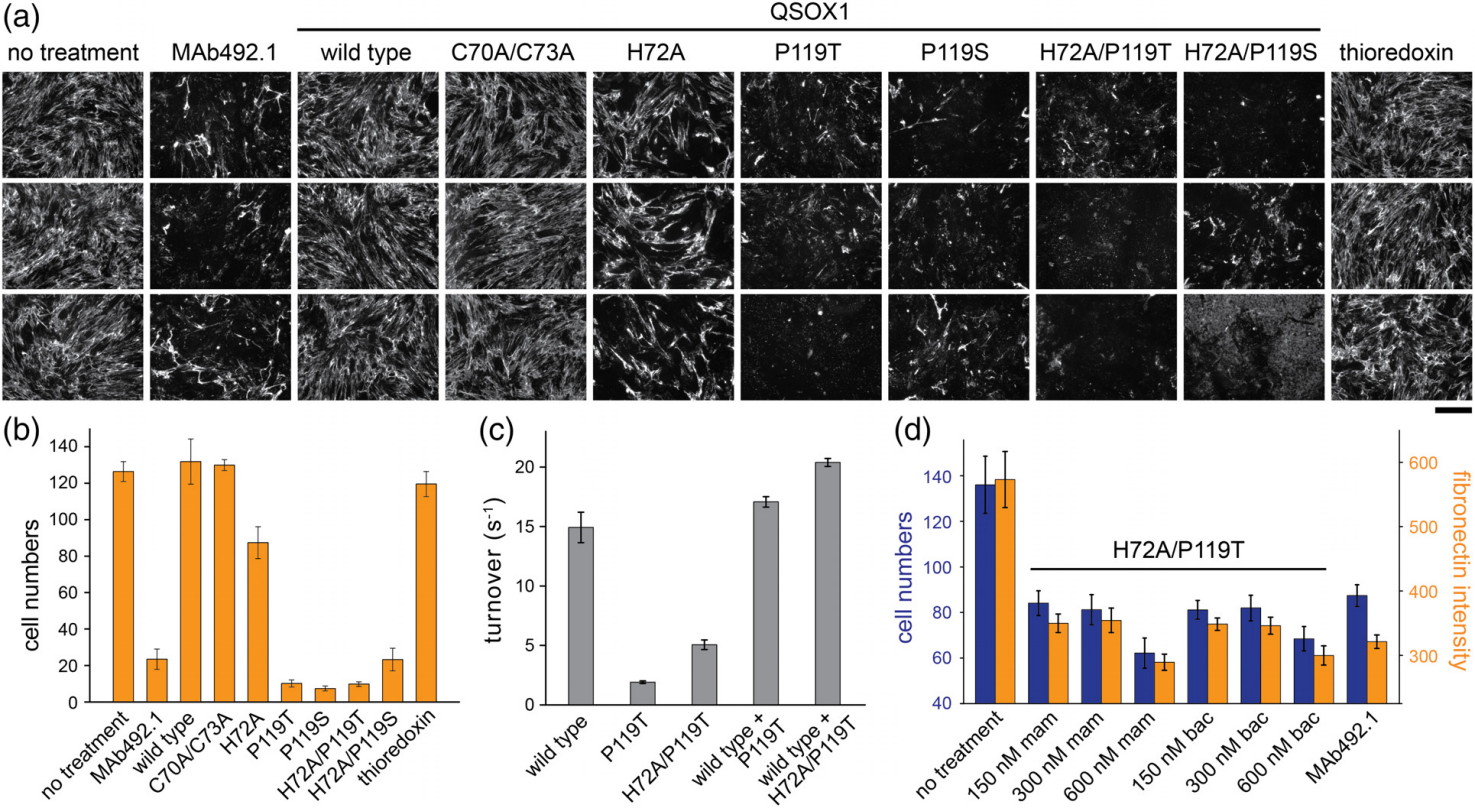
QSOX1 mutants disrupt ECM. (a) Representative images of fibronectin immunofluorescence in fibroblast cultures supplemented with 250 nM QSOX1 inhibitory antibody (MAb492.1) or 250 nM recombinant QSOX1 mutants produced in *E. coli* for 3 days. Scale bar (under the “thioredoxin” images) is 200 μm. (b) Quantification of remaining cells in the same fibroblast monolayers, counted by DAPI staining of nuclei in 15 × 10 fields of cells. Error bars are standard error of the mean. (c) QSOX1 mutants act independently in sulfhydryl oxidase assays. Turnover numbers were measured on 1 mM DTT with 100 nM of each indicated enzyme (i.e., a total of 200 nM when two mutants were present). Error bars are standard deviations. (d) Quantification of fibronectin and cell numbers 3 days after addition of the indicated concentrations of QSOX1 produced in bacteria or in mammalian cells. Error bars are standard deviations of three biological replicates. These results show that the H72A/P119T mutant has similar effects on fibroblast cultures whether it is produced in bacteria or mammalian cells. The severity of the disruption should not be compared with Panels (a) and (b) since differences in the cell passage number, confluence, growth rate, and other features of the primary fibroblast cells used in these experiments lead to variability in the degree of the effect after 3 days.

To test for effects in a system previously shown to require the activity of extracellular QSOX1 in the assembly of ECM,^17^ wild‐type QSOX1 and mutant proteins, all produced in *E. coli*, were supplied to the media of fibroblast cultures at a concentration about six‐fold higher than levels of secreted QSOX1.^17^ In this experiment, QSOX1 variants bearing the P119T or P119S mutations had a severely disruptive effect on ECM quantities and organization. As a measure of this effect, by 3 days after the addition of mutant enzymes, fibronectin fibers were nearly absent from the culture [Fig. 2(a)], and cell numbers were greatly reduced [Fig. 2(b)]. This phenomenon was similar to, but possibly more extreme than, the result of adding an inhibitory monoclonal antibody, MAb492.1, to block the activity of endogenous secreted QSOX1 [Fig. 2(a,b)].^17^, ^19^

Because the naturally secreted wild‐type enzyme was present together with the exogenously added mutant proteins, the mutants appear to act in a dominant negative fashion. In enzyme assays on the model substrate dithiothreitol (DTT), however, the wild‐type and mutant enzymes function additively [Fig. 2(c)], indicating that the mutants do not directly antagonize the catalytic activity of wild‐type QSOX1. Another potential mechanism for the deleterious effects of the *cis*‐proline mutants is competition with endogenous QSOX1 for extracellular enzyme localization sites. This explanation, however, is ruled out by the AXXA QSOX1 mutant, which lacks only the Trx active‐site cysteines and contains all other possible surfaces for interaction. Addition of the AXXA mutant to the fibroblast culture medium did not negatively affect ECM or cell adhesion [Fig. 2(a,b)], indicating that the *cis*‐proline mutants are disruptive due to the properties of their active sites. Lastly, we ruled out the deleterious effect being specific to QSOX1 mutants produced in bacteria by purifying H72A/P119T QSOX1 from mammalian cells. The H72A/P119T mutant behaved similarly over a range of concentrations regardless of the protein expression system [Fig. 2(d)].

### Redox properties of QSOX1 mutants

**Figure 3:**
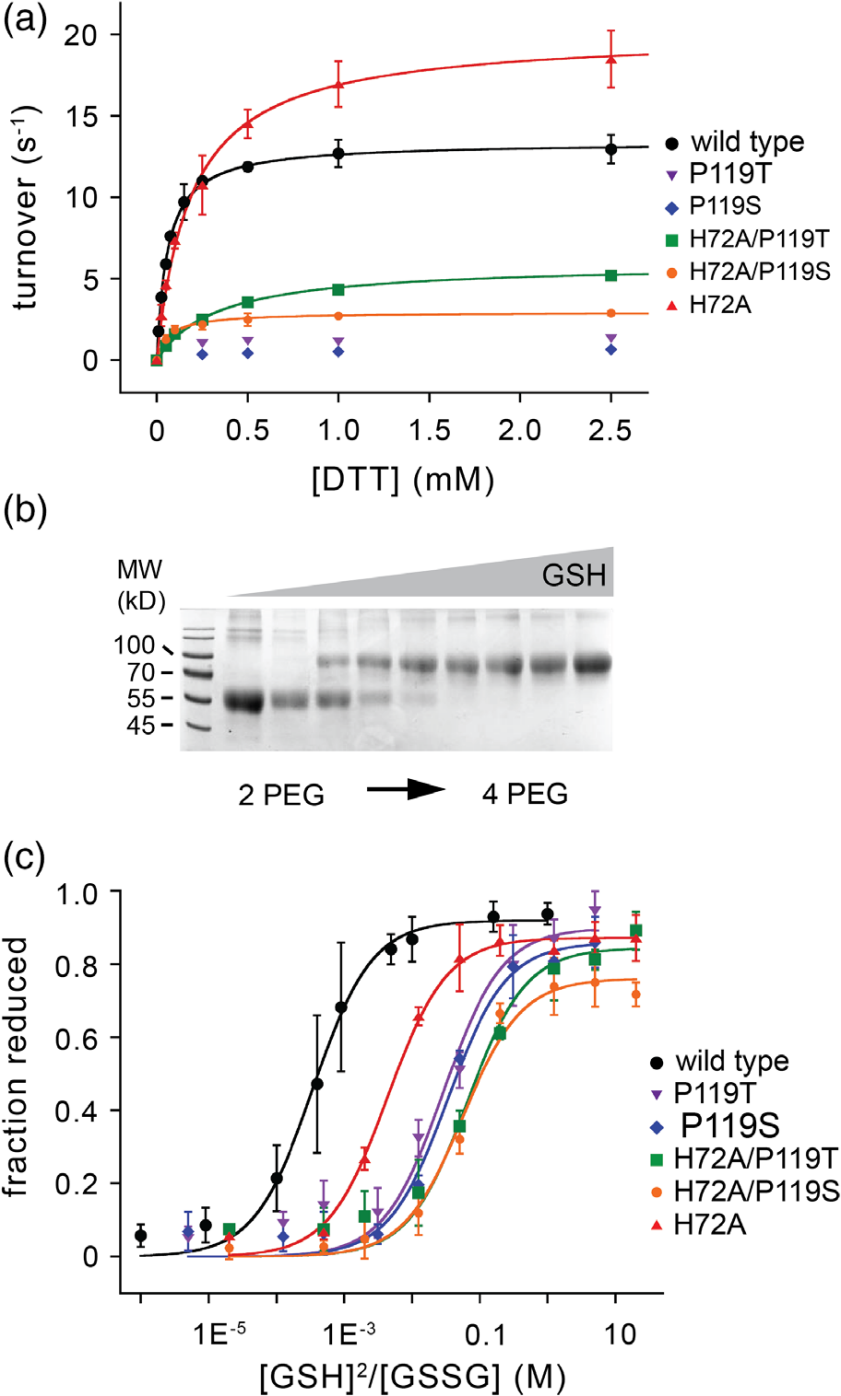
Effects of mutations on QSOX1 activity and active‐site redox potential. (a) Turnover numbers for QSOX1 mutants at a range of DTT concentrations were measured in an oxygen consumption assay. Error bars represent standard deviations from three measurements. *V*_max_ and *K*_M_ values are listed in Table 1. (b) Redox potential measurements for the CXXC motif in QSOX1(PDI) and its variants were obtained by equilibrating protein variants in buffer with a defined ration of reduced and oxidized glutathione. After modification of reactive cysteine thiolates with mal‐PEG, SDS‐PAGE was used to separate species with reduced versus oxidized active‐site disulfides. (c) Quantification of the reduced fraction of protein measure as shown in Panel B for wild‐type QSOX1(PDI) and various mutants. Error bars represent standard deviations from three measurements. For each protein, data were fit to the Hill equation to obtain redox potential values, which are presented in Table 1.

Given the above analysis, we consider it less likely that the *cis*‐proline QSOX1 mutants interfere directly with the disulfide‐bond forming activity of wild‐type QSOX1 and more likely that they engage in antagonistic redox reactions with ECM or cell‐surface targets in fibroblast cultures. To further characterize the redox properties of the QSOX1 variants, we measured their catalytic activities and redox potentials. The P119S and P119T variants showed severely decreased sulfhydryl oxidase activity [Fig. 3(a) and Table 1], comparable to mutation of the active‐site QSOX1 cysteines.^14^ Surprisingly, the H72A mutation increased *V*_max_ by about 1.5‐fold, but also increased the *K*_M_ value for DTT by about 3‐fold [Fig. 3(a) and Table 1]. The H72A mutation also partially rescued the *cis*‐proline mutations, restoring measurable sulfhydryl oxidase activity in the double mutants [Fig. 3(a) and Table 1]. Despite its high turnover on model substrates, the H72A mutant had a moderately disruptive effect on fibroblast cultures [Fig. 2(a,b)]. Moreover, the H72A mutation did not ameliorate the effects of *cis*‐proline mutations in the fibroblast culture assay [Fig. 2(a,b)]. Evidently, there is no correlation in this set of QSOX1 mutants between the degree of sulfhydryl oxidase activity on a model substrate and impact on ECM integrity.

**Table 1:** Enzymatic Parameters and Redox Potentials for QSOX1 Variants

QSOX1 variant or other protein	*V*_max_ (sec^−1^)	*K*_M_ DTT (μM)	Redox potential (mV)^a^	Qualitative effect on fibroblast cultures
wt	13.4 +/− 0.1	60 +/− 2	−138 +/− 4	None
C70A/C73A	NA	NA	NA	None
H72A	19.7 +/− 0.4	180 +/− 10	−170 +/− 1	Moderate
P119S	NA	NA	−196 +/− 2	Strong
P119T	NA	NA	−195 +/− 4	Strong
H72A/P119S	2.9 +/− 0.1	64 +/− 6	−201 +/− 2	Strong
H72A/P119T	5.7 +/− 0.1	300 +/− 20	−201 +/− 3	Strong
Trx	NA	NA	−270	None

a Determined in the context of QSOX1(PDI) fragment. NA is not applicable, because the relevant sites are absent or activities are below levels required for quantification.

The redox potentials of the *cis*‐proline and histidine mutants, and their combination, were next measured, using the reduced and oxidized glutathione couple. To avoid complications from electron transfer to the sulfhydryl oxidase module, these experiments were done in the context of the isolated tandem Trx domains, an enzyme fragment we refer to as QSOX1(PDI).^11^ The QSOX1(PDI) variants were equilibrated in redox buffers containing various ratios of reduced and oxidized glutathione, and mixtures were quenched by acid precipitation. Protein pellets were washed and then solubilized and neutralized in the presence of polyethylene glycol 5 kD containing a maleimide group (mal‐PEG). The mal‐PEG modifies cysteine thiolates and retards migration on denaturing electrophoretic gels to a comparable degree as the addition of 15 kD of ordinary polypeptide.^20^ QSOX1(PDI) naturally contains two unpaired cysteines, which become modified regardless of the redox state of the active site. Upon the background of these two mal‐PEG modifications, reduced active sites can be detected by an additional shift corresponding to about 30 kD per dithiol, enabling quantification of the fraction of reduced protein [Fig. 3(b)]. Redox potential values obtained for QSOX1(PDI) variants ranged from −138 mV for wild‐type QSOX1(PDI) to −201 mV for the H72A/P119S and H72A/P119T mutants [Fig. 3(c) and Table 1]. In contrast to the sulfhydryl oxidase activity of the QSOX1 mutants, which showed no relationship to the disruptive effect on ECM integrity, the redox potentials of the CXXC motif correlated with ECM disruption. Mutants with more negative redox potentials had a stronger effect on ECM structure and cell adhesive properties (Table 1). Notably, though redox potential in the QSOX1 mutant series was a relevant parameter, the addition of thioredoxin to cultured fibroblasts had no apparent effect [Fig. 2(a,b)], indicating specificity. Thioredoxin has a redox potential of −270 mV,^21^ lower than any of the QSOX1 variants tested.

### Structure of a QSOX1 *cis*‐proline mutant

**Figure 4:**
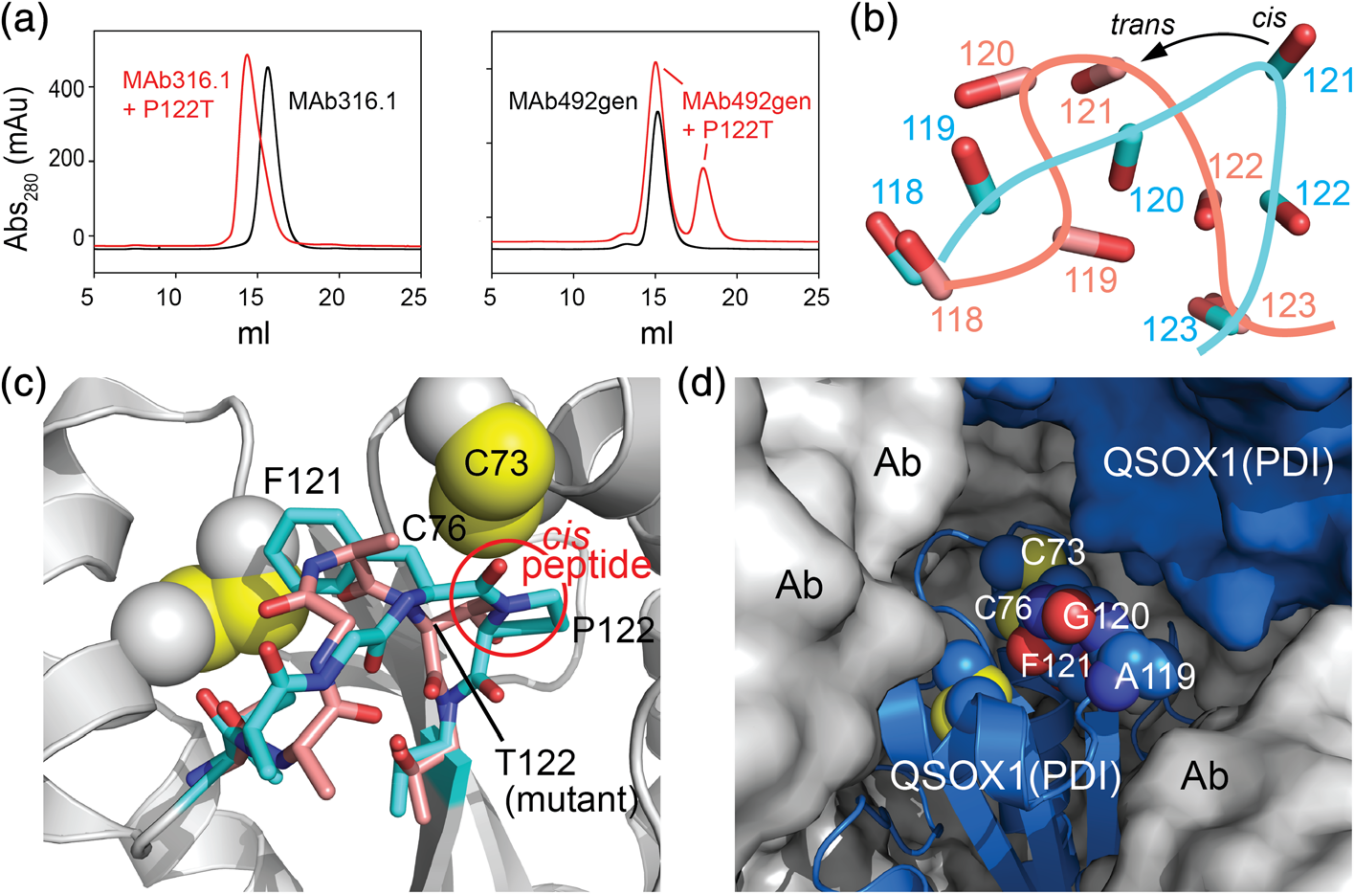
Structure of H75A/P122T mutant of murine QSOX1(PDI). (a) The MAb316.1 monoclonal antibody, but not the MAb492gen monoclonal antibody, binds the *cis*‐proline mutant of QSOX1(PDI) as shown by gel filtration. (b) Backbone carbonyls in the wild‐type and mutated loops are shown and labeled according to version (wild type: cyan; mutant: peach). Numbers are amino acid residue positions. The difference in orientation between the *cis* peptide bond carbonyl in the wild‐type enzyme and the *trans* form in the mutant is indicated by an arrow. (c) Wild‐type QSOX1(PDI), with the exception of the mutated loop, is shown in gray with yellow cysteine sulfurs, and the loops of the wild‐type and H75A/P122T versions are shown in cyan and peach color, respectively (nitrogens are blue and oxygens are red). The *cis* peptide bond in wild‐type QSOX1(PDI) is circled. (d) The mutated loop is in a solvent‐exposed channel in the crystal structure of QSOX1(PDI) H75A/P122T in complex with the antibody fragment (Ab). Surrounding molecules are shown in surface representation, with a symmetry‐related copy of QSOX1(PDI) H75A/P122T in blue. Loop residues and cysteines are shown in space‐filling representation.

To obtain structural insight into the origin of the changes in redox potential and the disruptive activity in cell culture, we determined a crystal structure of a QSOX1 *cis*‐proline mutant (Table 2). Mouse QSOX1(PDI) H75A/P122T, analogous to the human QSOX1 mutant H72A/P119T and representing one of the enzyme variants with the greatest deleterious effect, was crystallized in complex with an antibody Fab fragment after other approaches (see Methods) did not yield crystals. Two monoclonal antibodies that bind murine QSOX1 are available: MAb316.1 and MAb492gen.^19^, ^22^ The Fab (Fab316.1) used for crystallography was derived from MAb316.1, an antibody that inhibits murine QSOX1 by binding adjacent to, but not covering, the active‐site cysteines.^22^ Mab492gen, which inhibits QSOX1 by binding to the active‐site disulfide and *cis*‐proline region,^22^ was found not to bind the P122T mutant [Fig. 4(a)], suggesting that this region undergoes significant conformational changes upon mutation.

**Table 2:** Crystallographic Data and Refinement Parameters

Date collection	
Space group	*P*2_1_2_1_2_1_
Cell dimensions	
*a*, *b*, *c* (Å)	65.5, 112.7, 193.4
α, β, γ (°)	90, 90, 90
Complexes in asymmetric unit	2
Resolution (Å)	98–1.94 (1.94–2.02)
Measured reflections	106,284
Unique reflections	104,671 (8,728)
Completeness (%)	97.6 (82.5)
Redundancy	2.3 (2.3)
< *I*/σ*I* >	13.7 (1.6)
*R*_meas_	0.064 (0.79)
Refinement	
Resolution (Å)	48.9–1.94
Reflections in working set	104,667
Reflections in test set	5,215
*R*_work_/*R*_free_	0.194/0.231
Number of protein atoms	10,156
Number of water molecules	804
Mean B‐factor	42.1
Root mean square deviations	
Bond length (Å)	0.007
Bond angle (°)	0.882
Ramachandran plot	
Favored regions (%)	97.3
Additional allowed regions (%)	2.61
Disallowed regions (%)	0.08

Consistent with the loss of binding to MAb492gen, the structure of QSOX1(PDI) H75A/P122T revealed differences in backbone and side chain conformations in the region surrounding the proline‐to‐threonine mutation. Specifically, the pre‐proline peptide bond that had been in the *cis* configuration in the wild‐type enzyme was found in the *trans* configuration in the mutant [Fig. 4(b,c)], as previously observed for a proline‐to‐alanine mutation at the analogous position in DsbA.^23^ The outcome of this change is that the backbone carbonyl of F121, which would normally point toward the amino terminus of the active‐site helix and CXXC motif in position to interact with incoming substrate polypeptides,^15^, ^24^ is instead oriented in the opposite direction [Fig. 4(b,c)]. The side chain of the introduced threonine points roughly toward the active site, but it is not clear whether it might functionally substitute, in part, for the F121 carbonyl. Notably, the CXXC motif and *cis*‐proline loop are exposed to solvent within the crystal lattice and are not involved in crystal contacts [Fig. 4(d)], suggesting that the observed conformation represents the solution state of the protein.

The changes in the configuration of the pre‐proline peptide bond also propagate to other aspects of the structure. In particular, the conserved aromatic amino acid in the F/Y‐P–T motif is affected. An aromatic side chain at this position is universally observed in redox‐active domains of human PDI family proteins [Fig. 1(a)] and packs near the conserved alanine two residues before the conserved tryptophan upstream of the CXXC motif, as well as against a structural disulfide bond, when present [Fig. 4(c)]. In the H75A/P122T mutant, no electron density for the F121 side chain is seen in its normal buried position; instead, the main chain encroaches upon this space. An alternative location for the side chain is not evident from the electron density maps, suggesting that the phenylalanine becomes exposed to solvent and is flexible in the mutant. Supporting the notion of increased flexibility in this region upon mutation, both the absolute and relative temperature factors of the segment around Residue 122 are higher in the mutant than in the wild‐type protein. Specifically, the temperature factors for Residues 119–123 in wild‐type murine QSOX1(PDI) complexed with Fab 316.1 are only about 7% higher than the average for the first Trx domain,^22^ but the temperature factors for the comparable region of the H75A/P122T mutant are 47% higher than the corresponding domain average. This calculation for the mutant domain does not include the missing atoms for the phenylalanine side chain, which would presumably increase the temperature factors of this loop even further.

In addition to the structural changes in the *cis*‐proline loop observed in the H75A/P122T mutant, the active‐site CXXC was found in the reduced state in the crystal. The active‐site cysteines and *cis*‐proline loop are adjacent to one another, and their conformations may be coupled. Therefore, in principle, the differences in the *cis*‐proline loop could be a secondary effect of the different redox states of the proteins in the crystals. However, the changes to the loop noted above do not take place in the structure of reduced PDI (PDB ID 4EKZ), suggesting that they are due to the H75A/P122T mutations and not an inevitable consequence of active‐site disulfide reduction.

### Thermal stabilities and redox states of QSOX1 mutants

**Figure 5:**
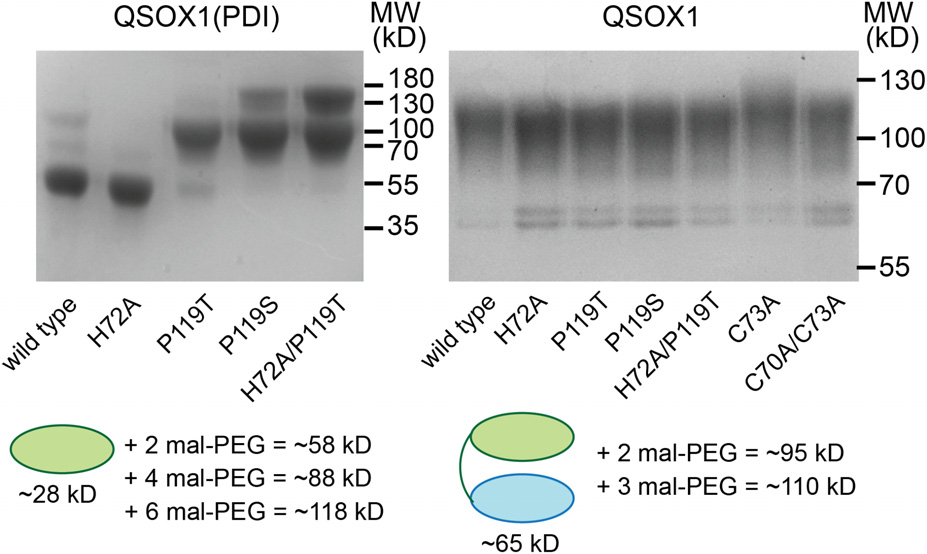
Redox states upon purification of QSOX1(PDI) and full‐length QSOX1 variants. Without the sulfhydryl oxidase domain, QSOX1(PDI) fragments bearing the P119T, P119S, and H72A/P119T mutations were obtained with reduced active sites (left gel). Additionally, the structural disulfides of H72A/P119T and P119S were found reduced to some extent. In the context of QSOX1 produced with its sulfhydryl oxidase domain in mammalian cells, the active sites of all relevant mutants were oxidized (right gel). The C73A mutant is a positive control for cysteine modification in this experiment. The proline and histidine mutants migrated similarly to the C70A/C73A mutant, which lacks active‐site cysteines, indicating that the cysteines in the proline and histidine mutants were not reactive to mal‐PEG and were apparently oxidized.

The solvent exposure of F121 in the murine QSOX1(PDI) H75A/P122T structure led us to hypothesize that the structural changes in the *cis*‐proline mutants might measurably shift the denaturation temperature. Therefore, a thermal shift assay was performed on the human QSOX1(PDI) variants. The depressed thermal resistance of the H72A/P119T mutant (Table 3) is consistent with removal of an aromatic side chain from a partially buried position as observed in the crystal structure. However, a lower denaturation temperature was not shared by other mutants: the H72A mutation increased resistance to thermal denaturation when introduced alone, whereas the P119T and P119S mutants showed similar denaturation temperatures as wild type (Table 3). In contrast, a second feature of the crystal structure was found to be common to the *cis*‐proline mutants, namely active‐site cysteines found in reduced form. To assess the redox states of the QSOX1(PDI) variants, the mal‐PEG procedure as described for redox potential measurements above was used without acid precipitation. The results showed that the wild‐type enzyme and the H72A variant were oxidized, whereas the P119T, P119S, and H72A/P119T mutants were reduced after purification from bacteria [Fig. 5(a)]. This observation is counterintuitive, since these mutants have redox potentials considerably lower than wild‐type QSOX1 [Fig. 3(c)]and are therefore expected to favor the oxidized state at equilibrium.^25^ These data suggest a kinetic barrier to oxidation of the proline mutants. Nevertheless, in the context of the intact QSOX1 enzyme, mutants were obtained in the oxidized state upon purification [Fig. 5(b)], such that all variants, with the exception of AXXA, were supplied in oxidized form in the fibroblast ECM assay. Irregularities in the kinetics of mixed disulfide formation and resolution may underlie both the substrate trapping behavior and the deleterious effect on fibroblast cultures of the *cis*‐proline mutants of DsbA and QSOX1, respectively.

**Table 3:** Thermal Shift Assay

QSOX1(PDI) variant	Midpoint of thermal denaturation (°C)
Wild type	62.4
H72A	67.9
P119T	63.2
P119S	63.2
H72A/P119S	56.2

## Discussion

The thioredoxin superfamily is represented in every known cellular organism and in many viruses as well. Member enzymes are found in a variety of intracellular compartments and participate in disulfide formation, isomerization, and reduction. Although many aspects of Trx protein sequences have diversified during their extensive evolution, certain amino acid positions and motifs are highly conserved across the superfamily. Most obviously, the active site, at the amino terminus of a surface helix, is typically a CXXC motif. However, flanking residues, such as the conserved tryptophan adjacent to the CXXC in sequence and the *cis*‐proline adjacent in space, are as highly conserved as the active‐site cysteines. Investigator‐driven mutation of these conserved positions in Trx proteins may serve two purposes. One is to probe the function of the conserved amino acids themselves (e.g., Ref. 26). The second is to manipulate the redox properties of the enzymes in desired ways to probe the roles and mechanisms of the enzymes in physiological settings.^6^, ^18^

It is with this second motivation that we mutated positions of QSOX1 that are conserved in PDI family members. Based on the retention of the *cis*‐proline from bacteria to human enzymes and the previous observation that mutation to threonine in *E. coli* DsbA introduced useful substrate‐trapping properties,^6^ we generated the analogous mutation in the human catalyst of disulfide bond formation QSOX1. The P119T mutation clearly introduced new properties into QSOX1, as demonstrated by the ability of this variant to disrupt fibroblast cultures, an ability not shared by exogenously added wild‐type QSOX1 or the catalytically inactive mutant AXXA [Fig. 2(a,b)]. It should be noted that the effects of the QSOX1 *cis*‐proline mutants in fibroblast cultures were not anticipated by activity assays *in vitro* [Fig. 3(a)]. Notably, proline‐to‐alanine mutations were previously made in the two redox‐active domains of PDI itself, but the properties of these mutants were tested only on catalytic activity *in vitro* and showed a simple loss of function.^27^ Just as the QSOX1 P119T and P119S mutants showed poor catalytic activity on model substrates in a sulfhydryl oxidase assay but differed dramatically from the catalytically inactive AXXA mutant in fibroblast cell cultures, PDI proline‐to‐threonine or ‐serine mutants may behave as antagonists in physiological contexts despite being loss‐of‐function mutants when measured by simple enzymatic turnover.

**Figure 6:**
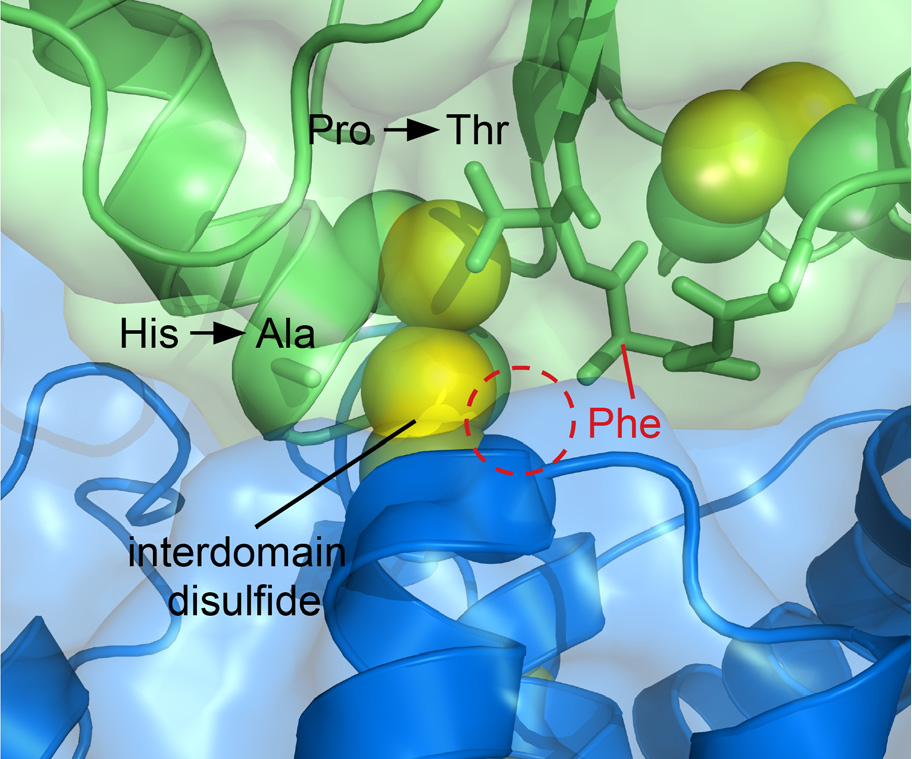
Model of the H75A/P122T mutant of murine QSOX1(PDI) docked onto the structure of the electron‐transfer intermediate of intact murine QSOX1. The QSOX1(PDI) region is green, and the sulfhydryl oxidase domain is blue. The loop from which the *cis*‐proline has been mutated to threonine is shown in stick representation. Despite the conformational differences in this loop compared to the wild‐type enzyme (see Fig. 4), there are no major steric clashes in the docked model, and flexibility in the loop may relieve the minor clashes. The missing phenylalanine side chain is represented by a dashed circle, and a red line points to the Cα atom of the residue. The absence of the histidine side chain due to the histidine‐to‐alanine mutation may make room for the phenylalanine during interdomain electron transfer in the H75A/P122T mutant.

It may seem surprising that the functional interaction between the PDI‐like and sulfhydryl oxidase domains was preserved in the QSOX1 mutants, despite the structural severity of the mutations [Fig. 4(b,c)]. Interestingly, a similar phenomenon was noted for the DsbA proline mutant, which remained a substrate for oxidation by DsbB.^23^ Structural information on the mode of interdomain redox communication within QSOX1 may justify the observations.^11^, ^15^ Few contacts are observed between the QSOX1 Trx domain *cis*‐proline loop and the sulfhydryl oxidase domain in structures showing these domains in redox communication.^11^, ^15^ In contrast, QSOX1, like DsbA, may make extensive interactions with its substrates using the *cis*‐proline loop.^28^ In the interdomain electron‐transfer interaction of QSOX1, analogous to electron transfer between DsbA and DsbB, the carbonyl from the *cis* peptide bond points toward the amino terminus of the helix bearing the redox‐active site in the sulfhydryl oxidase domain [Fig. 1(c)]. The F121 side chain is tucked inside the PDI‐like domain where it does not appear to contribute substantially to interdomain interaction. The minimal contacts between the *cis*‐proline loop and the sulfhydryl oxidase domain may be beneficial by not overly stabilizing the interdomain electron‐transfer intermediate, which should form only transiently.^29^ The minimal contacts are also what likely enable the QSOX1 PDI‐like module with a mutated loop to be oxidized. Docking of the structure of the murine QSOX1(PDI) H75A/P122T mutant onto the structure of an intact QSOX1 electron‐transfer intermediate mimic^11^ shows that the mutated loop is for the most part accommodated sterically (Fig. 6). The phenylalanine side chain, which did not show electron density in the structure of QSOX1 H75A/P122T and therefore does not appear in the model, might be expected to clash, but the flexibility of the loop may help minimize this problem.

This report suggests that certain tools related to Trx fold proteins may be transferred across kingdoms. A *cis*‐proline mutant of DsbA showed difficulty releasing substrates, whereas an analogous mutant of the human enzyme QSOX1, as demonstrated here, appeared to interfere with ECM formation and cell adhesion. In contrast to the situation with DsbA, however, ECM proteins are highly interconnected and cross‐linked and cannot readily be extracted from the ECM without reduction, which would destroy the putative mixed disulfides between the QSOX1 mutants and their targets in fibroblast cultures. Therefore, alternative approaches should be taken to pinpoint the proteins with which the QSOX1 mutants interact, as well as to confirm the mode of interaction. Since a gain of interesting properties was observed for both the bacterial DsbA proline‐to‐threonine mutant and the analogous human QSOX1 mutants, proline‐to‐threonine mutations in other mammalian PDI family members may produce useful functional variants.

## Materials and Methods

### Protein production

QSOX1 and QSOX1(PDI) variants expressed in bacteria were produced and purified as previously described.^11^, ^19^ For the H72A/P119T mutant produced in mammalian cells, the coding sequence corresponding to Residues 1–546 with an additional six histidines on the carboxy terminus was cloned into the pcDNA3.1 plasmid. The resulting construct was transiently transfected into HEK 293F cells (ThermoFisher). Cells were maintained in FreeStyle 293 medium and transfected using the PEI Max reagent (Polysciences Inc.) with a 1:3 DNA:PEI ratio (w/w). After 5 days, the cell suspension was spun for 10 min at 500 g, the supernatant was removed and spun for 30 min at 9500 *g*, and the supernatant from the second spin was filtered and purified by nickel‐nitrilotriacetic acid chromatography and size exclusion chromatography.

### Assays in fibroblast cultures

Human primary lung fibroblast cells (WI38) were plated 75,000 cells per well, in a 24‐well plate, 16 h prior to the addition of 250 nM recombinant proteins in 0.5 mL medium. This concentration is about six times the amount of endogenous QSOX1 that accumulates in confluent fibroblast cultures.^17^ After 3 days of incubation, cells were washed thrice with PBS containing calcium and magnesium (PBS++), fixed with 3.7% formaldehyde in PBS for 30 min at RT, washed thrice with PBS, and stained for 1 h at RT with a polyclonal rabbit α‐human fibronectin (DAKO) diluted 1:100 in 5% BSA in PBS‐T (100 μL per well). Afterwards, the cells were washed thrice with PBS‐T and incubated with DAPI stain and secondary α‐rabbit antibody conjugated to Alexa 488 (Abcam), diluted 1:200 in 5% BSA in PBS‐T with 100 μL per well for 30 min at RT. Samples were washed thrice more with PBS‐T, and 200 μL PBS was added to each well for imaging. DAPI‐stained nuclei and fibronectin fibers were imaged using an Olympus IX51 fluorescent microscope equipped with U‐MNIBA3 Fluorescence filter cube (excitation 470–495 nm, emission 510–550 nm for fibronectin imaging, and excitation 360–370 nm, emission 420 nm for DAPI imaging). Five 10× images were taken in each channel per well (15 images per treatment). Cells were quantified in every field of DAPI‐stained nuclei using ImageJ, and cell numbers were averaged for each treatment.

### Sulfhydryl oxidase assays

QSOX1 activity was measured based on the change in dissolved oxygen concentration using a Clarke‐type oxygen electrode (Hansatech Instruments). QSOX1 variants were diluted to 100 nM in 50 m*M* potassium phosphate buffer, pH 7.5, 65 m*M* NaCl, and 1 m*M* ethylenediaminetetraacetic acid (EDTA). Reactions were initiated by the injection of DTT to concentrations ranging from 50 μM to 2.5 m*M*, and turnover numbers were calculated from slopes of change in oxygen concentration as a function of time at 25°C.

### Redox potential

One micromolar QSOX1(PDI) variants were incubated at RT for 1 h in a volume of 1 mL containing a range of glutathione redox buffers. Redox buffers contained 5–100 μM oxidized glutathione (GSSG) and 10 μM–10 m*M* reduced glutathione (GSH) in 50 m*M* sodium phosphate buffer, pH 7, 200 m*M* NaCl, 1 m*M* EDTA, titrated to pH 7 with NaOH. The concentration of the GSH stock solution was determined using DTNB. The mixtures were quenched by the addition of trichloroacetic acid (TCA) to 20%, incubated at −20°C for 30 min, and left at 4°C ON. Precipitated protein samples were recovered by centrifugation (15 min at 4°C), and pellets were washed twice with ice‐cold acetone. After allowing pellets to air‐dry, the denatured protein samples were suspended in approximately 5 m*M* mal‐PEG in non‐reducing gel loading buffer before being analyzed by SDS‐PAGE using 12% acrylamide gels. The mal‐PEG was purified before use by gel filtration on a PD‐10 column to remove low‐molecular weight maleimides.^20^ Gel bands were quantitated using ImageLab. The fraction of reduced protein (i.e., functionalized with two additional mal‐PEG molecules) was plotted as a function of the redox buffer composition ([GSH]^2^/[GSSG]). Data fitting to the Hill equation was done using Origin to obtain the equilibrium constant *K*_ox_. Standard redox potentials were obtained by the Nernst equation with a standard redox potential value (E_0_) of −240 mV for GSH/GSSG.^30^

### Thermal denaturation

QSOX1(PDI) variants at a concentration of 10 μM were incubated with SYPRO Orange 5000× (Thermofisher) diluted 1:250 in a 96‐well PCR plate in 20 μL total volume in triplicate. Fluorescence data were collected using an Applied Biosystems Real‐Time PCR System using the fluorescein amidite channel with a temperature gradient of 1 min per 0.5°C from 24°C to 95°C. The peak of the first derivative of fluorescence with respect to temperature was taken as the transition midpoint (apparent denaturation temperature).

### Crystallization and structure solution

Numerous attempts were made in our laboratory to crystallize *cis*‐proline QSOX1 variants in the absence of antibody, without success. Among the constructs used in crystallization trials were full‐length rat QSOX1, human QSOX1(PDI), and mouse QSOX1(PDI), as well as methylated versions of these constructs. Antibody Fab fragments can aid crystallization due to their high solubility and ability to provide new surfaces for favorable protein–protein interactions, which are required for crystallization.^31^ Previously, formation of Fab complexes with QSOX1(PDI) modules allowed the crystallization of the module without reductive methylation, which was used to crystallize the wild‐type module in isolation.^11^ To generate the complex between murine QSOX1(PDI) H75A/P119T and Fab 316.1 that was analyzed in this study, the proteins were purified and then mixed at a 1:2 ratio of Fab to QSOX1(PDI) H75A/P119T for 30 min at 4°C to form complexes, which were purified from excess QSOX1(PDI) H75A/P119T by size‐exclusion chromatography. The complexes were concentrated to 10 mg/mL prior to crystallization. Crystals were grown by hanging‐drop vapor diffusion at 293 K by mixing 1 μL of protein complex solution with 1 μL of well solution containing 0.1 M sodium citrate, pH 6.0, 16% w/v PEG. Crystals were transferred to the same solution containing 25% w/v sucrose for freezing. Diffraction data were collected at the European Synchrotron Radiation Facility (ESRF) beamline ID23‐1 to 1.94 Å resolution from a crystal of space group P2_1_2_1_2_1_. The data set was processed and scaled using DENZO and SCALEPACK.^32^ Phases were determined by molecular replacement using Phaser.^33^ The MmQSOX1Trx–Fab316.1 complex structure^22^ without H75 and Residues 119–123 was used as a search model. Mutagenesis and model rebuilding were done using Coot.^34^ Refinement was performed using Phenix.^35^ Validation was done using MolProbity.^36^ Structure coordinates were deposited in the Protein Data Bank (code 6HF1).
